# Age dependent accumulation patterns of advanced glycation end product receptor (RAGE) ligands and binding intensities between RAGE and its ligands differ in the liver, kidney, and skeletal muscle

**DOI:** 10.1186/s12979-017-0095-2

**Published:** 2017-06-05

**Authors:** Myeongjoo Son, Wook-Jin Chung, Seyeon Oh, Hyosang Ahn, Chang Hu Choi, Suntaek Hong, Kook Yang Park, Kuk Hui Son, Kyunghee Byun

**Affiliations:** 1 Department of Anatomy and Cell Biology, Graduate School of Medicine, Gachon University, Incheon, 21936 Republic of Korea; 2 Functional Cellular Networks Laboratory, Lee Gil Ya Cancer and Diabetes Institute, Gachon University, Incheon, 21999 Republic of Korea; 3 Department of Cardiovascular Medicine, Gachon University, Incheon, 21999 Republic of Korea; 4 Gachon Cardiovascular Research Institute, Gachon University, Incheon, 21999 Republic of Korea; 5 Department of Thoracic and Cardiovascular Surgery, Gachon University Gil Medical Center, Gachon University, Incheon, 21565 Republic of Korea; 6 Laboratory of Cancer Cell Biology, Department of Biochemistry, School of Medicine, Gachon University, Incheon, 21999 Republic of Korea

**Keywords:** RAGE, RAGE ligands, AGEs, Aging, Macrophage activation

## Abstract

**Background:**

Much evidence indicates receptor for advanced glycation end products (RAGE) related inflammation play essential roles during aging. However, the majority of studies have focused on advanced glycation end products (AGEs) and not on other RAGE ligands. In the present study, the authors evaluated whether the accumulation of RAGE ligands and binding intensities between RAGE and its ligands differ in kidney, liver, and skeletal muscle during aging.

**Results:**

In C57BL/6 N mice aged 12 weeks, 12 months, and 22 months, ligands accumulation, binding intensities between RAGE and its ligands, activated macrophage infiltration, M1/M2 macrophage expression, glyoxalase-1expression, and signal pathways related to inflammation were evaluated. The RAGE ligands age-associated accumulation patterns were found to be organ dependent. Binding intensities between RAGE and its ligands in kidney and liver increased with age, but those in skeletal muscle were unchanged. Infiltration of activated macrophages in kidney and liver increased with age, but infiltration in the skeletal muscle was unchanged. M1 expression increased and M2 and glyoxalase-1 expression decreased with age in kidney and liver, but their expressions in skeletal muscle were not changed.

**Conclusion:**

These findings indicate patterns of RAGE ligands accumulation, RAGE/ligands binding intensities, or inflammation markers changes during aging are organs dependent.

**Electronic supplementary material:**

The online version of this article (doi:10.1186/s12979-017-0095-2) contains supplementary material, which is available to authorized users.

## Background

The aging process can be described as a universal, intrinsic, progressive accumulation of deleterious changes in cells and tissues that increase morbidity and lead to death [1]. According to the recent theory of oxidation-inflammation, chronic oxidative and inflammatory stress conditions explain the aging process [2].

Several studies have focused on the role played by receptor for advanced glycation end products (RAGE) on aging, because RAGE is known inducer of inflammation and oxidative stress.

RAGE belongs to the immunoglobulin superfamily of cell surface molecules and has an extracellular region containing one V-type immunoglobulin domain and two C-type immunoglobulin domains [3, 4]. The extracellular portion of the receptor is followed by a hydrophobic transmembrane-spanning domain and then by a highly charged, short cytoplasmic domain that is essential for intracellular RAGE signaling [3, 4].

RAGE has several ligands, such as, advanced glycation end products (AGEs), proinflammatory S100/calgranulin family members, and high motility group box 1 protein (HMGB1) [5, 6]. RAGE is also a signal transduction receptor for amyloid β [7] and endogenous phospholipids such as lysophosphatidic acid [8].

After binding these ligands, RAGE activates an inflammation-related signaling cascade involving nuclear factor-(NF)κB, ERK (extracellular signal-regulated kinase) 1/2, p38 MAPK (mitogen-activated protein kinases), JNK (c-Jun N terminal kinases), PKC (protein kinase C), Rac/Cdc42, and TIRAP and MyD88 (adaptor proteins for TLR 2 and 4) [5, 6].

Because AGEs accumulates in organs, such as, kidney [9], liver [10], brain [11], and skeletal muscle during aging [12], researches have tended to investigate the role played by the AGEs-RAGE pathway during aging. The AGEs-RAGE pathway is a primary contributor to kidney aging [13]. The accumulation of AGEs and a progressive decline in renal function during aging may induce the release of inflammatory mediators and the generation of reactive oxygen species (ROS) [10, 11]. Furthermore, this accumulation starts before a clinical decrease in kidney function is evident, and is one of the characteristic features of kidney aging [14, 15].

Although much evidence indicates that RAGE-related inflammation and oxidative stress participate in the aging process, the majority of studies on the topic have been focused only AGEs and not on other RAGE ligands [13–15]. In fact, few studies have addressed the roles of these other RAGE ligands in different organs in same animals.

Therefore, we sought to determine whether the accumulation patterns of the RAGE ligands which are AGEs, HMGB1, and S100β and the binding intensities between RAGE and its ligands in kidney, liver, and skeletal muscle are age-dependent.

## Methods

### Animals

A total of 18 male C57BL/6 N mice were used and kidney, liver, and skeletal muscle were extracted from young (12 weeks old, *n* = 6), middle-aged (12 months old, *n* = 6), and old (22 months old, *n* = 6) mice. The skeletal muscle was collected from adductor profundus of mouse hind limb. All animals were examined daily for signs of injury or illness by trained persons. When tissue samples were collected no abnormality was evident. This study was approved by Lee Gil Ya Cancer and Diabetes Institute of Gachon University, and conducted in strict accordance with the guidelines issued by our Institutional Animal Care and Use Committee (Approval number; LCDI–2015–0080).

### Sample preparation

#### Protein preparation

Kidney, liver, and skeletal muscle proteins were extracted using the EzRIPA lysis kit (ATTO, Tokyo). All tissues were homogenized with lysis buffer plus proteinase inhibitor and phosphatase inhibitor and briefly sonicated 10 times for 10 s in a cold bath sonicator. After centrifuging at 14,000 x g for 20 mins at 4 °C, supernatant phases were collected and protein concentrations were determined using a Bicinchoninic acid assay kit (Thermo Scientific, IL, USA).

#### RNA isolation and cDNA synthesis

Total RNA in kidney, liver, and skeletal muscle from young, middle-aged, and old mice were isolated using a Trizol reagent (Invitrogen, CA, USA) according to the manufacturer’s instructions. All tissues were homogenized in ice using a disposable pestle in 1 ml of Trizol reagent. Homogenized tissues were added to 0.2 ml of chloroform (Amresco, OH, USA), mixed, and centrifuged at 12,000 x g for 15 mins at 4 °C. Aqueous phases were collected, placed in cleaned tubes, mixed with 0.5 ml of isopropanol, and centrifuged using the same conditions. Isolated RNA was then washed with 70% ethanol, and dissolved in 30 μl of DEPC treated water. To perform quantitative real time polymerase chain reaction (qRT-PCR), 1 μg of total RNA was subjected to complementary DNA (cDNA) synthesis using the PrimeScript 1st strand cDNA Synthesis Kit (TAKARA, Japan).

#### Paraffin block tissue slides

Mice were anesthetized with Rumpun (2.5 mg/kg) and Zoletil (500 mg/kg). Kidney, liver, and skeletal muscle tissues from young, middle-aged, and old mice were fixed in 4% paraformaldehyde (Sigma-Aldrich, MO, USA) overnight and placed in an automatic dehydration machine (Leica ASP-300 S). Tissues were dehydrated in a series of steps, that is, with 90% ethanol for 3 × 1 h, followed by 100% ethanol for 2 × 2 h. Tissues were then cleared with 100% xylene for 3 × 1.5 h, embedded in warmed paraffin, and paraffin embedded tissue blocks were sectioned at 10 μm.

### Immunohistochemistry

Macrophage infiltration was identified by the expression of Iba1 in kidney, liver and skeletal muscles. Paraffin was removed by zylene and washed three times for 10 mins each in phosphate-buffered saline (PBS). Endogenous peroxidase was blocked by 0.3% hydrogen peroxide in PBS, followed by three times rinses with PBS and we then blocked for 1 h with normal serum. The tissues sections were incubated with anti-Iba1 antibody (see in Additional file 1: Table S1) overnight at 4 °C and allowed to react with 3,3′-diaminobenzidine (DAB) from standard ABC kit (Vector Laboratories).

### Enzyme-linked Immunosorbent assay (ELISA) assay

#### Indirect ELISA assay

AGE, HMGB1, and S100β levels in kidney, liver and skeletal muscle tissues in young, middle-aged and old mice were measured by an Indirect ELISA. Coating solution mixture (0.6% sodium bicarbonate and 0.3% sodium carbonate in distilled water) were incubated onto 96-well plates overnight at 4 °C. Protein samples were mixed with 5% skim milk containing 0.1% Triton x-100 in phosphate-buffered saline (TPBS) and then incubated overnight at 4 °C. Unbound proteins were removed by washing with TPBS and incubated with anti-AGE, anti-HMGB1, and anti-S100β antibodies for 6 h at room temperature (see in Additional file 1: Table S1). Unbound antibodies were removed by washing with TPBS, and then incubated with horseradish peroxidase (HRP) conjugated anti-rabbit antibody for 2 h at room temperature. After washing out unbound HRP conjugated antibody, color was developed by incubating samples with 3,3′,5,5′-tetramethylbenzidine (TMB) for 15 mins. The reaction was stopped by adding 100 μl of 2 M H_2_SO_4_ to each well, and absorbance was then measured at 450 nm using an ELISA plate reader (VERSA Max, Molecular Devices).

#### Sandwich ELISA assay

RAGE-RAGE ligand bindings in kidney, liver, and skeletal muscle in young, middle-aged and old mice were determined by sandwich ELISA. A 96-well plate was coated by anti-RAGE antibody with coating solution mixture overnight at 4 °C. Unbound anti-RAGE antibody was removed with washing with TPBS. To reduce non-specific binding, proteins were mixed with 5% skim milk containing TPBS and incubated overnight at 4 °C. Unbound proteins were removed by washing repeatedly with TPBS. Anti-RAGE antibody binding samples were treated with anti-AGE, anti-HMGB1, and anti-S100β antibodies for 2 h at room temperature (Additional file 1: Table S1). After washing with TPBS, bound proteins were incubated with HRP conjugated anti-rabbit secondary antibody for 2 h at room temperature. After washing off unbound HRP conjugated secondary antibody, color was developed by incubating samples with TMB for 15 mins. Reactions were terminated by adding same volumes of H_2_SO_4_ and absorbance was measured using the ELISA plate reader.

### Quantitative real time polymerase chain reaction (qRT-PCR) analysis

To quantify genesglyoxalase 1 (GLO-1) level, we used quantitative real time polymerase chain reaction (qRT-PCR). Briefly, 300 ng cDNA, 5 μl ROX plus SYBR green premix (TAKARA) and 0.4 μM forward and 0.4 μM reverse primers (Additional file 1: Table S2) were mixed for each gene, and levels of gene expression were then determined using a CFX384 Touch™ Real-Time PCR Detection System (Bio-Rad).

### Immunoblotting analysis

To estimate the M1/M2 macrophage infiltration and levels of inflammatory proteins including NFκB and interleukin-1β (IL-1β), 30 μg of proteins per lane were separated by 4–12% NuPAGE Bis-Tris gel (Thermo Scientific) electrophoresis. Proteins were then transferred to polyvinylidene fluoride (PVDF) membranes at 200 mA for 2 h. Non-specific binding was blotted with 5% skim milk. Membranes were then treated with primary antibodies (Additional file 1: Table S1), washed with Tris-buffered saline containing 0.1% Tween 20 (TTBS) three times and incubated with secondary antibodies (Additional file 1: Table S1). Membranes were developed by enhanced chemiluminescence (Thermo Scientific) on a LAS-4000 analyzer (GE healthcare).

### Statistical analysis

Given the small sample size, non-parametric analysis was used. Statistical analyses were performed using SPSS version 22 (IBM Corporation, NY, USA). The Kruskal-Wallis test was used to compare groups, and the Mann-Whitney U test was used for multiple comparison.

*P* values of <0.05 were deemed significant. All results are expressed as means ± standard deviations.

## Results

### Age increased RAGE ligands levels in kidney and liver but not in skeletal muscle

**Fig. 1:**
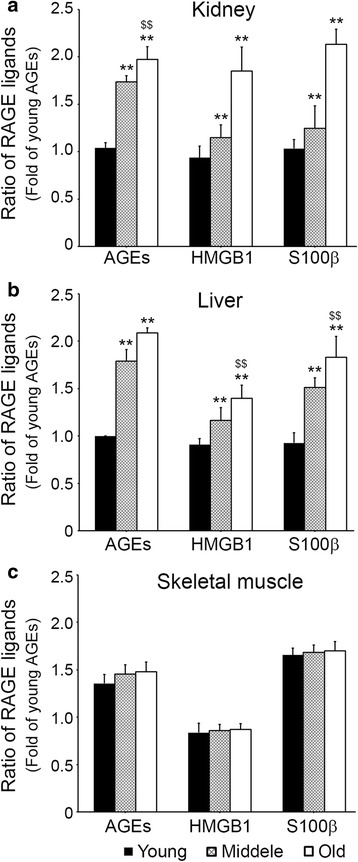
Age-related RAGE ligands expression difference in kidney, liver and skeletal muscle. The level of RAGE ligands including AGEs, HMGB1, and S100β in the **a** kidney, **b** liver and **c** skeletal muscle of young, middle-aged, old mice were validated by In-direct ELISA. Ratios represented in the graphs represent fold levels of AGEs versus young mice. **; *p* < 0.01 versus young mice, $$; *p* < 0.01 versus middle-aged mice

The AGEs accumulation in the kidney of young group was significantly lower than in the middle-aged group, and there was difference between middle-aged and old group in the kidney. The accumulation of HMGB1 and S100β in kidney of the three age groups was significantly increased with age (Fig. 1a). In liver, AGEs accumulation was increased by aging. HMGB1 and S100β accumulations in liver were significantly lower in the young group than in the middle-aged group, and HMGB1 and S100β levels in liver were different between the middle-aged and old groups (Fig. 1b). No difference in skeletal muscle levels was found between the three age groups (Fig. 1c).

### Binding intensities between RAGE and its ligands increased with age in kidney and liver but not in skeletal muscle

**Fig. 2:**
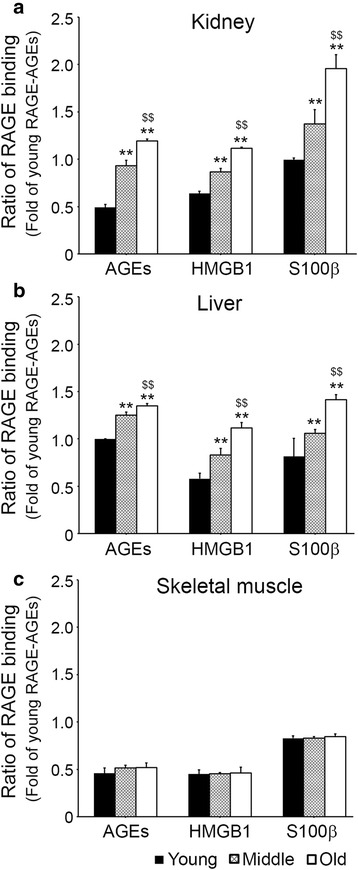
Age-dependent binding affinities between RAGE with RAGE ligands in kidney, liver and skeletal muscle. The binding levels of RAGE-RAGE ligands, which are, AGEs, HMGB1 and S100β in **a** kidney, **b** liver and **c** skeletal muscle of young, middle-aged, and old age mice were determined by Sandwich ELISA. Ratios in graphs represented fold of RAGE-AGEs binding levels versus young mice. **; *p* < 0.01 versus young mice, $$; *p* < 0.01 versus middle-aged mice

In kidney, binding intensities between RAGE and AGEs significantly increased with age and the RAGE/HMGB1 and RAGE/S100β binding intensities increased with age (Fig. 2a). In additional, RAGE/AGEs, RAGE**/**HMGB1, and RAGE/S100β binding intensities were increased with aging in liver (Fig. 2b). However, in skeletal muscle, no intergroup difference was observed for any of binding intensities between the three ligands (Fig. 2c).

### Increased infiltration of activated macrophages and expressions of M1 and M2 in tissues by age

**Fig. 3:**
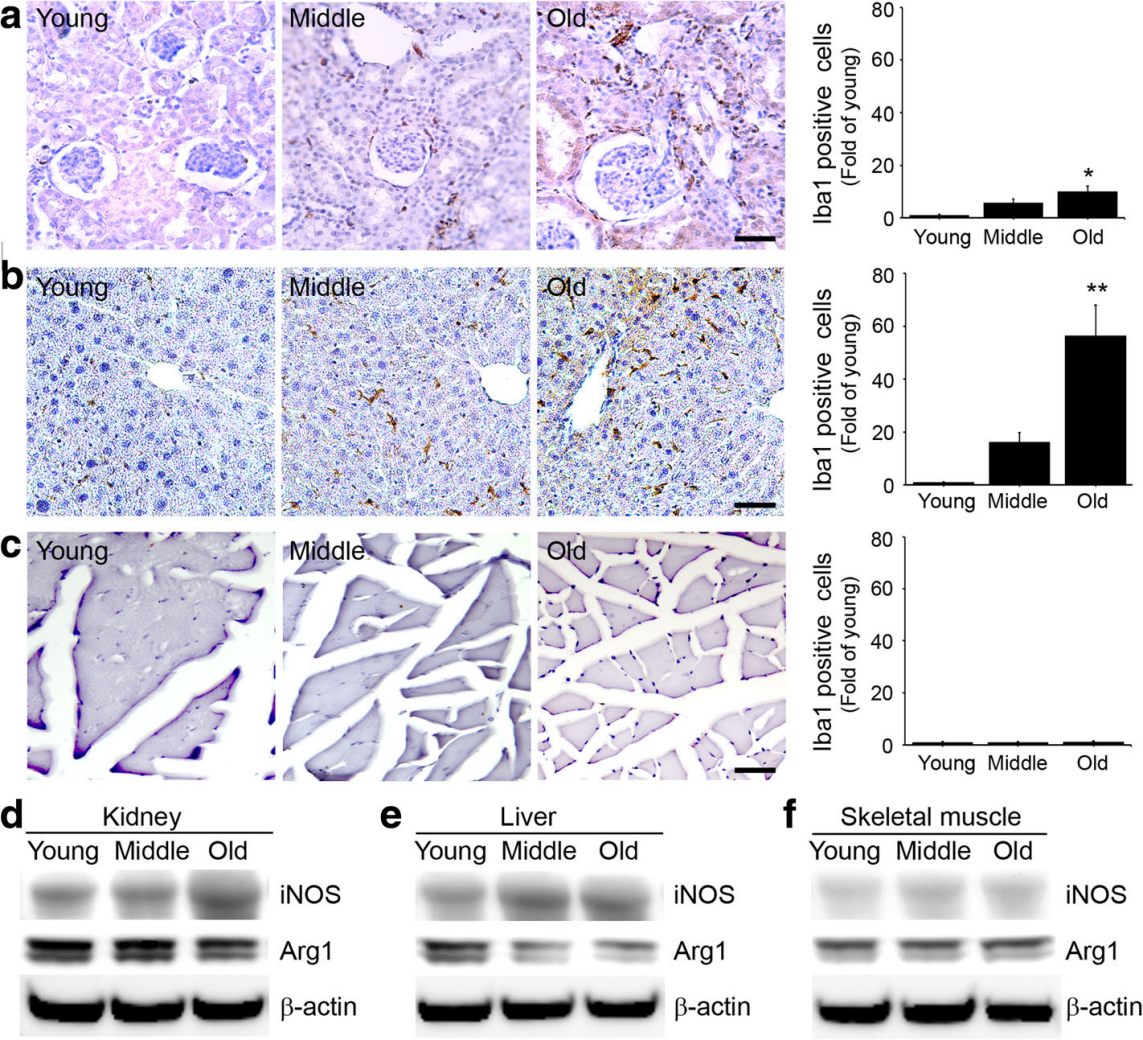
Age-related expressions of total, M1, and M2 macrophages in kidney, liver and skeletal muscle. The Expression of total, M1 and M2 macrophages were determined by Immunohistochemistry and Immunoblotting. The level of Iba1 represented total macrophage expression in **a** kidney, **b** liver and **c** skeletal muscle. The level of iNOS represented M1 macrophage expression in **d** kidney, **e** liver and **f** skeletal kidney. The level of arginase 1 (Arg 1) represented M2 macrophage expression in **d** kidney, **e** liver and **f** skeletal kidney. Ratios in graphs are folds levels versus young mice. Scale bar = 100 um, *; *p* < 0.05 and **; *p* < 0.01 versus young mice

In kidney and liver, infiltrations of activated macrophage (Iba1) into tissues increased significantly with age (Fig. 3a, b). However, no intergroup difference was found for activated macrophage infiltration into skeletal muscle (Fig. 3c). In kidney and liver, the expressions of M1 (iNOS) significantly increased with age (Fig. 3d, e). However, the expression of M1 in skeletal muscle was similar in the three groups (Fig. 3f). In kidney and liver, the expression of M2 (Arg1) in all three groups significantly decreases with age (Fig. 3d, e). However, the expression of M2 in skeletal muscle was similar in the three groups (Fig. 3f).

### Glyoxalase-1 levels decreased and inflammatory protein levels increased in kidney and liver but not in skeletal muscle with age

**Fig. 4:**
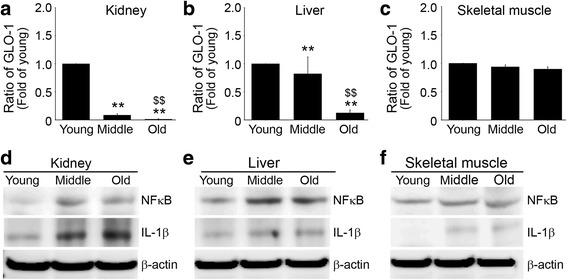
Age-related changes in the levels of GLO-1 in kidney, liver and skeletal muscle. Levels of Glo-1 in **a** kidney, **b** liver and **c** skeletal muscle of the young, middle-aged, and old groups were validated by qRT-PCR. Ratios in graphs represented fold levels versus young mice. Expressions of NF_K_B and IL-1β were determined by immunoblotting in **d** kidney, **e** liver and **f** skeletal muscle. **; *p* < 0.01 versus young mice, $$; *p* < 0.01 versus middle-aged mice

In kidney and liver, GLO-1 levels were significantly different in the three groups decreased with age. In addition, there was statistical difference middle-aged and old group (Fig. 4a, b). However, in skeletal muscle no age-related differences were observed (Fig. 4c).

In kidney and liver, NFκB and IL-1β levels increased with age (Fig. 4d, e), but in skeletal muscle, no differences were observed (Fig. 4f).

## Discussion

The present study shows; (1) the age-related accumulation patterns of RAGE ligands (AGEs, HMGB1, and S100β) are organ dependent; (2) binding intensities between RAGE and its ligands in kidney and liver increased with age, but binding intensities in skeletal muscle were not changed by aging; (3) infiltrations of activated macrophage into kidney and liver were increased with age, but infiltrations into skeletal muscle were not changed; (4) M1 expression was increased and M2 expression was decreased by age in kidney and liver, but M1 expression in skeletal muscle was unchanged; (5) GLO-1 expressions in kidney and liver were decreased by age, but unchanged in skeletal muscle; and (6) the activation of inflammation related signal pathway in kidney and liver increased with age, but in skeletal muscle they remained unchanged.

Several studies have shown that the AGEs-RAGE pathway is related to aging of humans and animals [16, 17]. In addition, many studies have shown AGEs accumulates in tissues during aging. The liver is a site for clearance and catabolism of circulating AGEs but can also be a target organ for AGEs [10, 18]. AGEs are removed and metabolized by the kidney, but the kidney is also a site for AGEs accumulation and AGEs-associated damage [19]. It is well known that older adults exhibit increased collagen cross-linking and AGEs deposition in skeletal muscle [12]. Thus, we considered that accumulation of AGEs in liver, kidney, and skeletal muscle would be more prominent than in other tissues during aging.

In the present study, AGEs accumulations in kidney in the middle-aged and old groups were significantly higher than in the young group, however no difference was observed between the middle-aged and old groups. These results suggest AGEs accumulation in kidney reaches a maximum level even in middle-age. AGEs are generated endogenously by glycation, and this process is enhanced by ROS or hyperglycemic conditions or by ingestion of exogenous AGEs in food [13]. Physiological glycation state is regulated by a balance between the formation and clearance of AGEs [20], and this balance is maintained in part by glycation state, host defense machinery, including anti-glycation enzymes (e.g., glyoxalase), and kidney filtration function (glomerular filtration rate), which excretes AGEs and AGEs precursors from the body [13, 20].

The glomerular changes in C57B6 mice begin at 18 to 20 months of age, before recognizable tubulointerstitial changes, and these progressively increase to death at ~30 months [21, 22]. Although glomerular function might be preserved until 20 months [21, 22], it is known that GLO-1 levels in kidney decrease with age [23]. Many previous studies have shown AGEs accumulation in kidney linearly increases with age. The present study shows that AGEs accumulation peaked during middle-age in our animal model. Though we did not check glomerular function in the present study, we did observe GLO-1 expression decreased with aging, which concurs with other studies [23]. We speculate that decreasing GLO-1 activity by aging plays an important role in the renal accumulation of AGEs, because AGEs accumulation in kidney might accelerate before middle-age when glomerular filtration function is preserved [21, 22].

We found that AGEs accumulation in liver was significantly increased by aging, whereas GLO-1activity decreased, which agrees with other studies [24]. Age-related AGEs accumulation in skeletal muscle has been previously reported in an animal model [25], but in the present study, AGEs accumulation was unchanged by aging. Even GLO-1 levels were not changed in skeletal muscle. Other studies have shown an increasing pattern of AGEs accumulation in skeletal muscle by aging used 33-month rats as the old groups [25]. In the present study, we used 22-month mice, which could explain the discrepancy between results, and suggests AGEs accumulation in the skeletal muscle might accelerate after 22 months in mice.

Unlike AGEs, the age-related accumulation patterns of other RAGE ligands, such as, HMGB1 and S100β, have not been in different organs. However, the effects of the accumulations of HMGB1 and S100β during aging have been in the context of brain aging. In particular, the expression of S100 protein is increased in the aging brain [26]. It has also been reported that the distribution of HMGB1 appears to be altered in the aged brain [27]. More specifically, HMGB1 is downregulated in neurons in the aged brain, but it is upregulated in astrocytes, which suggests HMGB1 plays different roles in different types of brain cells and structures [27].

In the present study, HMGB1 and S100 β accumulation in kidney increased with age. In liver, there was no difference between the middle-aged and old groups in HMGB1 and S100β accumulation. In skeletal muscle, the accumulations of both ligands were not changed by aging.

Although RAGE ligand age-related accumulation patterns differed in kidney, liver, and skeletal muscle, binding intensities between RAGE ligands and RAGE increased with age in kidney and liver. These patterns paralleled age-related inflammation signal pathway activation, activated macrophage infiltration, M1 increases, and M2 decreases.

Ligand binding with RAGE triggers ROS increases, activates NADPH oxidase, increases the expressions of adhesion molecules, and upregulates inflammation through NFκB and other signaling pathways [18, 28]. Furthermore, activation of NFκB results in increased RAGE expression, thereby prolonging NFκB activation [29]. In addition, RAGE expression occurs in an inducible manner and is upregulated at sites where its ligands accumulate [29].

In the present study, we measured binding intensities between RAGE and its ligands rather than RAGE expression. Binding intensities between RAGE and its ligands in the liver and kidney were increased by aging, regardless of age-related ligand accumulation. The patterns of binding intensities between RAGE and its ligands in the liver and kidney matched NFκB and IL-1β activations, which suggests that binding intensity between RAGE and its ligands in the liver and kidney is a more important factor of age-related inflammation and oxidative stress than absolute ligand accumulations.

A previous study showed that the RAGE pathway can enhance macrophage migration [30] and promote proinflammatory mediator production, such as, those of IL-1β, IL-6, and TNF-α [31]. The RAGE/NF-κB pathway not only predominantly induces macrophages to secrete inflammatory cytokines but also induces M1 polarization [32], and M1 macrophages are Th1-biased and considered to be pro-inflammatory and most notably express TNFα and IL-6 [33]. The majority of the macrophages found in sites of inflammation in inflammatory diseases are considered to be M1 macrophages [33]. By contrast, M2 macrophages are Th2-biased and are thought to play more diverse roles, namely in anti-inflammatory pathways, tissue remodeling and wound healing [33].

The patterns of binding intensities between RAGE and its ligands in liver and kidney were similar to patterns of M1 expression increase and M2 expression decrease with age in liver and kidney, which suggests local tissue inflammation was increased by aging in liver and kidney and that the RAGE pathway plays an important role in the aging process. On the contrary, the aging process of skeletal muscle appears to be different from those of liver or kidney. The binding intensity between RAGE and its ligands, activation of inflammatory signal pathway**s,** macrophage activation, and M1 polarization were not changed by aging in skeletal muscle, which suggests the RAGE pathway and inflammation induced by RAGE pathway do not play a main role in skeletal muscle aging. In aged animal skeletal muscle, AGEs cross-linking collagen was increased and those collagen made muscle stiff [11, 25]. Taken together, we speculate that mechanical property changes, such as, increased muscle stiffness caused by collagen fiber cross linking by AGEs might be more important than RAGE induced inflammation during skeletal muscle aging.

The main limitation of this study was that we did not measure ROS levels in tissues directly, however many studies have been shown that ROS levels increase during aging in many tissues. Our study shows NFκB increased with age in kidney and liver, and the NFκB signal pathway is known to be importantly related to ROS generation.

## Conclusion

Our study shows binding intensities between RAGE and its ligands increased during aging and that these increases occur in parallel with activated macrophage infiltration, macrophage polarization, and inflammation signal pathway activation in the kidney and liver. However, skeletal muscle did not show any age-related changes in RAGE ligands accumulation, binding intensities, or inflammation marker changes, which suggest aging processes differ in different organs.

## Additional file

Additional file 1: Table S1. Antibodies list used for Immunohistochemistry, ELISA and immunoblotting. **Table S2.** List of primers for Quantitative polymerase chain reaction (qRT-*PCR*). (DOC 46 kb)

## Abbreviations

AGEs: Advanced glycated endproducts
GLO-1: Glyoxalase-1
HMGB1: High-mobility group box 1
RAGE: Receptor of AGE
ROS: Reactive oxygen species
