# Supplementary material for: Age dependent accumulation patterns of advanced glycation end product receptor (RAGE) ligands and binding intensities between RAGE and its ligands differ in the liver, kidney, and skeletal muscle
Source: Immun Ageing. 2017 Jun 5;14:12. doi: 10.1186/s12979-017-0095-2 (PMC5460364; doi:10.1186/s12979-017-0095-2)
Supplement: Additional file 1: Table S1. — Antibodies list used for Immunohistochemistry, ELISA and immunoblotting. Table S2. List of primers for Quantitative polymerase chain reaction (qRT-PCR). (DOC 46 kb) [file 12979_2017_95_MOESM1_ESM.doc]

**Additional file 1**

Table S1. Antibodies list used for Immunohistochemistry, ELISA and immunoblotting

| Antigen (host) | Company | Cat. No | Application | | |
| --- | --- | --- | --- | --- | --- |
|  |  |  | ELISA | IB | IHC |
| β-actin (rabbit) | abcam | ab8227 | - | 1:4,000 | - |
| NF-kB p65 (rabbit)  (phosphor S529) | abcam | ab97726 | - | 1:1,000 | - |
| IL-1β (rabbit) | santa-cruz | sc7884 | - | 1:100 | - |
| AGE (rabbit) | abcam | ab23722 | 1:1,000 | - | - |
| RAGE (goat) | abcam | ab7764 | 1:200 | - | - |
| HMGB1 (rabbit) | abcam | ab128129 | 1:1,000 | - | - |
| S100 β (rabbit) | abcam | ab52642 | 1:500 | 1:500 | - |
| Iba1 (goat) | abcam | ab5076 | - | - | 1:1,000 |
| Peroxidase labeled anti-rabbit IgG | Vector | PI1000 | 1:1,000 | 1:5,000 | - |

IHC; Immunohistochemistry, IB; Immunoblotting, ELISA; Enzyme-linked immunosorbent assay

Table S2. List of primers for Quantitative polymerase chain reaction (qRT-PCR)

| Gene | | Primers sequence |
| --- | --- | --- |
| *GAPDH* | Forward | 5'-CGT CTT CAC CAC CAT GGA AGA-3’ |
|  | Reverse | 5'-CGG CCA TCA CGC CAC AGT TT-3’ |
| *GLO-1* | Forward | 5'-GGGAAGTGGAGTAAAACAATGGT-3’ |
|  | Reverse | 5'-CTTGGCTAGAAGCAAGCGTGA-3’ |
